# Whole-Genome Sequences of *Burkholderia pseudomallei* Isolates Exhibiting Decreased Meropenem Susceptibility

**DOI:** 10.1128/genomeA.00053-17

**Published:** 2017-04-06

**Authors:** Erin P. Price, Melissa Laird Smith, Ellen E. Paxinos, Luke J. Tallon, Lisa Sadzewicz, Naomi Sengamalay, Robert W. Baird, Bart J. Currie, Derek S. Sarovich

**Affiliations:** a Global and Tropical Health Division, Menzies School of Health Research, Charles Darwin University, Darwin, Northern Territory, Australia; b Centre for Animal Health Innovation, Faculty of Science, Health, Education and Engineering, University of the Sunshine Coast, Sippy Downs, Queensland, Australia; c Pacific Biosciences, Menlo Park, California, USA; d Institute for Genome Sciences, University of Maryland School of Medicine, Baltimore, Maryland, USA; e Department of Infectious Diseases and Northern Territory Medical Program, Royal Darwin Hospital, Darwin, Northern Territory, Australia; f Pathology Department, Royal Darwin Hospital, Darwin, Northern Territory, Australia

## Abstract

We report here paired isogenic *Burkholderia pseudomallei* genomes obtained from three patients receiving intravenous meropenem for melioidosis treatment, with post-meropenem isolates developing decreased susceptibility. Two genomes were finished, and four were drafted to improved high-quality standard. These genomes will be used to identify meropenem resistance mechanisms in *B. pseudomallei*.

## GENOME ANNOUNCEMENT

*Burkholderia pseudomallei* is a Gram-negative soil- and water-borne bacterium that causes the tropical infectious disease melioidosis. Melioidosis severity ranges widely, with the most serious form of disease, septic shock, resulting in fatality in up to 95% of untreated cases (1). *B. pseudomallei* is intrinsically resistant to many antibiotics commonly used in sepsis treatment, limiting treatment options and often resulting in progressive disease when not diagnosed (2, 3). In Australia, where melioidosis mortality rates have decreased to approximately 10% (4), ceftazidime is the mainstay of intravenous therapy for melioidosis, with meropenem usually reserved for life-threatening sepsis requiring intensive care therapy (5). We recently identified three Australian blood culture-persistent patients in whom decreased meropenem sensitivity has been observed (D.S. Sarovich, J. R. Webb, M. C. Pitman, L. Viberg, M. Mayo, R. W. Baird, B. J. Currie, E. P. Price, unpublished data); this is the first time that this phenomenon has been reported. Identifying the molecular mechanisms underpinning decreased meropenem susceptibility in *B. pseudomallei* is vital in detecting resistance emergence toward this life-saving antibiotic. The genome of another clinical isolate of *B. pseudomallei* with imipenem resistance, a related carbapenem, has recently been described (6).

Three paired isogenic *B. pseudomallei* isolates were examined in this study (Table 1). The first isolates were sensitive toward meropenem, whereas the latter isolates had decreased sensitivity according to MIC testing. The six isolates were extracted as previously described (7), with the addition of RNase treatment. Genomic DNA was subjected to Illumina paired-end HiSeq2000 whole-genome sequencing (Macrogen Inc., Geumcheon-gu, Seoul, Republic of Korea) to ~55× coverage. In addition, PacBio single-molecule real-time sequencing was conducted on the PacBio RS II instrument (Institute for Genome Sciences, Baltimore, MD, USA) to ~13× coverage using 20-kb SMRTbell libraries and P6C4 chemistry.

**TABLE 1:** Isogenic *B. pseudomallei* strains sequenced in this study

Strain pair^a^	Multilocus sequence type^b^	No. of contigs (status)^c^	Genome size in bp (% GC content)	GenBank and/or SRA accession no.
MSHR3763	36	2 (finished)	7,437,013 (67.9)	CP017052, CP017053; SRS1143371
MSHR4083	36	2 (finished)	7,437,071 (67.9)	CP017050, CP017051; SRS1143382
MSHR5864	975	2 (IHQD^d^)	7,317,380 (68.1)	CP017048, CP017049
MSHR6755	975	2 (IHQD^d^)	7,300,607 (68.1)	CP017046, CP017047
MSHR6522	437	3 (IHQD^d^,^e^)	7,285,806 (68.2)	MECZ00000000
MSHR7929	437	2 (finished)	7,248,498 (68.2)	CP017044, CP017045

a The latter strain from each pair has decreased sensitivity to meropenem.

b Based on the scheme at http://pubmlst.org/bpseudomallei.

c Assembly definitions according to Chain et al. (14). IHQD, improved high-quality draft.

d Trimming of chromosome 1 was not possible due to overlap issues.

e The third contig aligns to the end of chromosome 1.

PacBio genomic data were assembled using HGAP.3 (8) (MSHR strains 3763, 4083, and 5864), Celera Assembler version 8.2 (9) (MSHR strain 6522), and Celera Assembler version 8.3 (MSHR strains 6755 and 7929). Assemblies were reorganized relative to the closed *B. pseudomallei* K96243 genome (10) (GenBank accession no. CP009538 and CP009537) with the assistance of progressiveMAUVE (11), followed by error-correction with the Illumina reads using iCORN2 (12). For MSHR strains 3763 and 4083, SPANDx version 3.1 (13) was used to identify a handful of remaining errors in the assemblies, which were manually corrected and verified by repeat analysis in SPANDx. All variants were also confirmed by comparison of the MSHR strain 3763 and MSHR strain 4083 assemblies using Illumina-only assemblies generated by MGAP.

The development of *B. pseudomallei* resistance toward meropenem is of great concern, as this drug is one of a handful of efficacious antimicrobials for melioidosis treatment. Meropenem resistance is especially concerning given that this antibiotic is used to treat the most severe melioidosis cases in Australia and some other melioidosis-endemic regions. Treatment failure in such cases must be rapidly identified in the clinical setting to enable clinicians to alter therapy in close-to-real time. The six genomes reported in this study will be used to search for genetic variants imparting decreased meropenem susceptibility in *B. pseudomallei*.

### Accession number(s).

The genome sequences of the *B. pseudomallei* isolates reported here have been deposited in GenBank under the accession numbers listed in Table 1.
